# Protective Effect of Polysaccharides from *Inonotus obliquus* on Streptozotocin-Induced Diabetic Symptoms and Their Potential Mechanisms in Rats

**DOI:** 10.1155/2014/841496

**Published:** 2014-06-30

**Authors:** Bao-zhong Diao, Wei-rong Jin, Xue-jing Yu

**Affiliations:** ^1^Department of Pharmaceutical Preparations, Liaocheng People's Hospital and Liaocheng Clinical School of Taishan Medical University, Liaocheng, Shandong 252000, China; ^2^Department of Endocrinology, The First Affiliated Hospital of Jiamusi University, Jiamusi, Heilongjiang 154002, China

## Abstract

The present study aimed to evaluate the therapeutic effects of polysaccharides from *Inonotus obliquus* (PIO) on streptozotocin- (STZ-) induced diabetic symptoms and their potential mechanisms. The effect of PIO on body weight, blood glucose, damaged pancreatic *β*-cells, oxidative stresses, proinflammatory cytokines, and glucose metabolizing enzymes in liver was studied. The results show that administration of PIO can restore abnormal oxidative indices near normal levels. The STZ-damaged pancreatic *β*-cells of the rats were partly recovered gradually after the mice were administered with PIO 6 weeks later. Therefore, we may assume that PIO is effective in the protection of STZ-induced diabetic rats and PIO may be of use as antihyperglycemic agent.

## 1. Introduction

Diabetes mellitus is a metabolic disorder affecting carbohydrate, fat, and protein metabolism. Long-standing diabetes is prone to various complications which include cardiac, kidney, and eye problems [1]. More effective and safer treatment modalities for diabetes mellitus need to be investigated. PIO is a mushroom habiting in the cold latitudes of Europe and Asia, which was used as traditional Chinese medicine for a long time. In the last decade, several studies have reported biological activities of PIO such as anticancer, antioxidation, anti-inflammatory, and antihyperglycemic activities and enhancement of immunity [2–5]. Up to now, however, no detailed investigation has been carried out on the effective constituents of PIO for antihyperglycemic activities. At the same time, the limited natural resources of* I. obliquus* limit its role as therapeutic agent for diabetes mellitus.

Many studies have shown that polysaccharides from PIO possessed clear antioxidant activities [6, 7]. There is growing evidence that free-radical-mediated oxidative processes are involved in the pathogenesis of diabetic complications and oxidative stress is implicated in cardiac dysfunction, leading to heart failure in diabetes [8]. In the present study, the purpose is to focus on the isolation and hypoglycemic properties of polysaccharide fractions from fermented mushroom of PIO for seeking new natural functional ingredients used in food and pharmaceutical industry to alleviate the diabetes mellitus.

## 2. Materials and Methods

### 2.1. Fermented Mushroom of* Inonotus obliquus*

A strain of PIO was used in this study. The seed was grown at 27°C for 7 days on PDA slants (1,000 mL 20% potato extract liquid +20.0 g dextrose +20.0 g agar). 10 pieces of the mycelia of* Inonotus obliquus* were transferred from a slant into each Erlenmeyer flask containing 50 mL seed medium with the sterilized self-designed cutter. The culture was incubated at 27°C-28°C on a rotary shaker at 180 rmp for 8 days.

### 2.2. Preparation of Polysaccharides from* I. obliquus* (PIO)

Dried mycelium of* I. obliquus* was extracted with distilled water (600 mL) at 121°C for 2 h. After cooling and filtration, the extract was concentrated to one-tenth of the volume and precipitated with 4 vol of 95% ethanol at 4°C for 24 h. Polysaccharides were precipitated from resuspended extracts using 75% ethanol followed by exhaustive dialysis with water for 48 h, giving thewater-soluble polysaccharide of PIO.

### 2.3. Animals

Healthy male adult Wistar rats (2 months old and weighing 200 ± 20 g) were used in the study. This study was performed in accordance with the Guide for the Care and Use of Laboratory Animals. Care was taken to minimize discomfort, distress, and pain of the animals. Experimental diabetes was induced by intraperitoneal (i.p.) injection with freshly prepared solution of STZ (Sigma, USA) dissolved in citrate buffer (pH 4.5) at the dose of 35 mg/kg body weight. Only rats with blood glucose concentration more than 240 mg/dL were considered diabetic and used for the study. Glucose level was assessed by using enzymatic glucose oxidase peroxidase commercially available kit method, 72 h after STZ induction. The rats with blood glucose concentration more than 240 mg/dL were considered diabetic and used for the study.

### 2.4. Treatment Schedule and Experimental Protocol

Forty hyperglycemic rats were selected and allocated equally into 4 groups and administered orally saline, PIO (10 mg/kg/d), PIO (20 mg/kg/d), and PIO (30 mg/kg/d), respectively. The other 10 normal rats were administered orally with the saline and used as the control group.

Body weight of all animals was recorded on 0, 1st, 2nd, 3rd, 4th, 5th, and 6th week of treatment. Blood of all animals was collected through retroorbital route initially and on 6th week of treatment to measure the serum glucose levels. Then, the rats were sacrificed. The blood sample was allowed to clot for 20 minutes at refrigerator temperature. The blood samples were then shifted to clean centrifuge tubes. Lithium heparin was added to obtain plasma. The withdrawn blood was separated by centrifugation at 4000 rpm for 10 minutes to obtain serum. The serum was stored in freezer until analysis. The liver was dissected out for the measurement of IL-1*β* and TNF-*α*. The pancreas was reserved for pathological histology using hematoxylin and eosin (H&E) staining.

### 2.5. Measurement of IL-1*β* and TNF-*α* Level in Liver

The liver was dissected out for the measurement of hepatic glycogen. The liver TNF-*α* and IL-1*β* were measured using a commercial enzyme-linked immunosorbent assay (ELISA) kit (Shanghai Jinma Biological Technology, Inc., China) following the manufacture's instruction.

### 2.6. Measurement of Lipid Profile

Total cholesterol (TC), triglycerides (TAG), low-density lipoprotein (LDL) cholesterol, and high-density lipoprotein (HDL) cholesterol were determined in the serum samples using commerciallyavailable kits (Shanghai Jinma Biological Technology, Inc., China).

### 2.7. Measurement of Glucose Metabolizing Enzymes

The liver homogenate was used to assess metabolizing enzymes. Glutamic oxaloacetic transaminase (GOT), glutamic pyruvic transaminase (GPT), and lactate dehydrogenase (LDH) were measured using commerciallyavailable kits (Shanghai Jinma Biological Technology, Inc., China).

### 2.8. Estimation of the Total Antioxidant Activity

The total antioxidant status (TAOS) of hepatic tissue was determined by the way introduced by Laight et al. [9]. The increase in absorbance at 405 nm was measured by using a microplate reader (Shanghai Xunda Medical Technology, Inc., China).

### 2.9. Statistical Analysis

All data were analyzed by a one-way analysis of variance, and the differences between means were established by Duncan's multiple-range test. The data represents means and standard deviations. The significant level of 5% (*P* < 0.05) was used as the minimum acceptable probability for the difference between the means.

## 3. Results and Discussion

The objective of this study was to investigate whether the polysaccharides from* I. obliquus* (PIO) could produce hypoglycemic activity in STZ-induced diabetic rats. STZ is an antibiotic extracted from* Streptomyces achromogenes* and is diabetogenic due to a selective cytotoxic action upon pancreatic *β*-cell [10]. In the present investigation, STZ injected rats exhibit clinicopathological features including biochemical, oxidative, and metabolic changes. These changes were halted in PIO treated animals.

**Figure 1:**
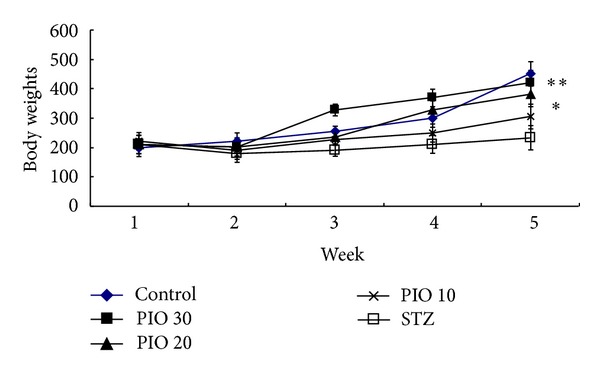
Effect of PIO administration on diabetic rats' body weight. Values represent the mean ± SEM. *P < 0.05 versus STZ group.

Many studies have shown an association between hyperglycemia and decreased body weight of diabetic animals [11]. As shown in Figure 1, the STZ-treated animals had significantly reduced body weight than the control rats (*P* < 0.01). When compared with STZ-treated animals, the body weight gains were significantly increased in groups of PIO-treated animals (*P* < 0.05; *P* < 0.01) in a dose-dependent manner.

STZ in the experimental diabetic model leads to defective glucose oxidation and causes hyperglycemia [12]. Our study is in agreement with this report. The blood glucose level in normal rats remained constant for six weeks and was significantly (*P* < 0.01) lower than those of streptozotocin-induced diabetic rats (Table 1). Upon treatment with PIO for six weeks, the blood glucose levels of all diabetic rats were markedly diminished in a dose-dependent manner, suggesting that DPM is a potent therapeutic agent against diabetes.

The hypoglycemic mechanisms of many polysaccharides are closely related to their antioxidant activity [13]. Hence, it is plausible that the hypoglycemic effect of PIO may be due to the effect on alleviating oxidative stress. The TAOS is an indication of O_2_^−^ and other oxidant species. We measured TAOS activity as an indirect indication of the formation of O_2_^−^ and other oxidant species. The results of hepatic TAOS are shown in Table 2. The STZ treatment increased TAOS. TAOS in the PIO-20- and PIO-30-treated groups were significantly lower than those in the STZ-treated group (*P* < 0.05 and *P* < 0.01, resp.).

It has been observed that over 75% of early deaths in diabetes are related to coronary artery disease caused by abnormal lipid metabolism, which often leads to altered lipid profile of the victim [14]. Lipid peroxidation is one of the characteristic features of chronic diabetes. The increased free radicals produced may react with polyunsaturated fatty acids in cell membranes leading to lipid peroxidation. It will, in turn, result in the elevated production of free radicals [15]. In the present experiment, significantly increased lipid peroxidation products were observed in STZ-induced diabetic rats. Treated with PIO-20 and PIO-30 for 6 weeks, LDL level was reduced (*P* < 0.05), whereas HDL cholesterol was increased (*P* < 0.05) (Table 3). These results further confirm that there is a strong correlation between oxidative stress and diabetes occurrence.

It was suggested that the STZ-induced weight loss in animal was the result of protein wasting in a situation of unavailability of carbohydrate for utilization as an energy source [11]. In diabetes, cytoplasmic enzymes such as GOT, GPT, and LDH pass into blood plasma and their activities in serum increase [16]. In the present study, oral treatment of PIO-20 and PIO-30 significantly (*P* < 0.05) restored the altered glycoprotein components of diabetic rats in a dose-dependent manner (Table 4).

**Figure 2:**
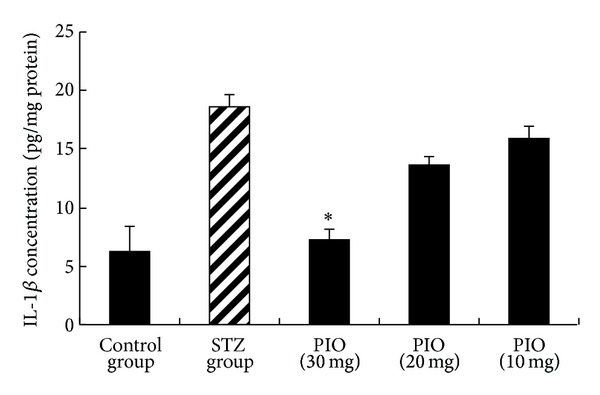
Effect of PIO on IL-1*β* level. Values represent the mean ± SEM. *P < 0.05 versus STZ group.

**Figure 3:**
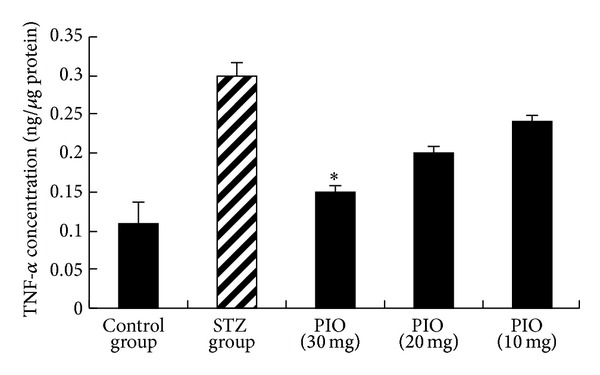
Effect of PIO on TNF-*α* level. Values represent the mean ± SEM. *P < 0.05 versus STZ group.

A chronic inflammation may have a role in the pathogenesis of metabolic disorders [17, 18]. Prospective studies have identified proinflammatory cytokines as predictors of diabetes [19]. TNF-*α* was the first proinflammatory cytokine implicated in pathogenesis of obesity-related insulin resistance and diabetes [20] and studies conducted with IL-1*β* antagonism beneficial effects on glycated hemoglobin and *β*-cell function [21]. Therefore, the effect of PIO on TNF-*α* and IL-1*β* production was determined by ELISA. In comparison to STZ group (Figure 2), treatment with PIO-30 resulted in a marked decrease in IL-1*β* levels (*P* < 0.05). In addition, PIO-30 suppressed STZ-induced TNF-*α* production (*P* < 0.05) (Figure 3).

**Figure 4:**
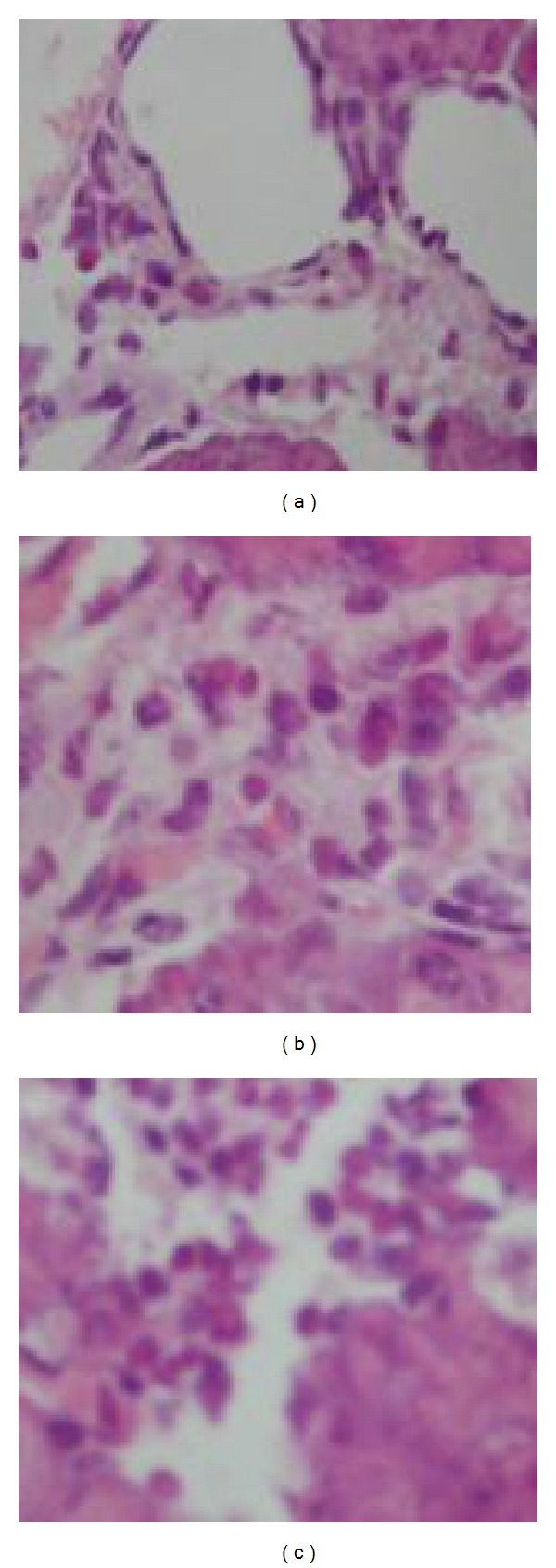
Islet cell death and replication represented by hematoxylin-eosin. The islet cells of diabetic rat of STZ treatment (b) showed extensive cell lysis, representing loss of plasma membrane with condensed nuclei and dissolved cytoplasm in wide intercellular spaces. In contrast, the islet cell of PIO-fed rat (c) was partly recovered. (c) Islet from a normal rat.

STZ is a compound commonly used to induce diabetes in rodents. The mode of its action is mediated through the induction of severe damages to the *β*-cells [22]. The protective effect of PIO against the damages to *β*-cells induced by STZ toxicity was investigated. Selective destruction of pancreatic *β*-cells by STZ in the experimental diabetic model was observed (Figure 4(b)). We observed focal necrosis, congestion in central vein, and infiltration of lymphocytes in the pancreas of STZ. Such lesions were considerably diminished by PIO-30 (Figure 4(c)). Further, *β*-cells structure of the PIO rats appeared normal. This indicated that PIO could significantly protect the *β*-cells from STZ-induced cell damage. This result strongly supported the therapeutic potential of PIO against diabetes.

In summary, we have shown that PIO has therapeutic effects against diabetes via multiple pathways. It displays antioxidant actions, hypolipidemic activity, and protects the pancreas from the diabetes induced injuries in STZ-treated rats. Therefore, PIO may provide a valuable therapeutic option against diabetes.

## Figures and Tables

**Table 1:** Effect of PIO on blood glucose levels in STZ-hyperglycemic rats.

Different groups	Blood glucose (mmol/L)
STZ group	22.2 ± 2.2
PIO (30 mg) group	10.1 ± 3.2*
PIO (20 mg) group	15.5 ± 2.0
PIO (10 mg) group	18.6 ± 3.0
Control group	5.9 ± 1.2

Values are means ± SEM; *n* = 10. *P < 0.05 versus STZ group.

**Table 2:** Effect of PIO on TAOS activity (*μ*M L-ascorbate).

Different groups	TAOS activity (*μ*M L-ascorbate)
Control group	28.40 ± 3.10
STZ group	81.33 ± 5.32
PIO (10 mg) group	72.24 ± 2.78*
PIO (20 mg) group	65.30 ± 3.31*
PIO (30 mg) group	56.30 ± 4.34**

Values are shown as means ± SEM; *P < 0.05 versus STZ group; **P < 0.01 versus STZ group.

**Table 3:** Effect of PIO on changes in the levels of serum lipid profile.

Lipid profile mmol/L	Control group	STZ group	PIO (10 mg) group	PIO (20 mg) group	PIO (30 mg) group)
LPO	8.6 ± 0.51	13.3 ± 3.8	9.3 ± 0.31	8.9 ± 0.40	7.4 ± 0.48**
Cholesterol	4.30 ± 0.79	10.40 ± 0.85	8.30 ± 0.50	7.30 ± 0.80	4.34 ± 0.80
Triglycerides	0.80 ± 0.11	1.23 ± 0.10	0.98 ± 0.14	0.81 ± 0.09	0.78 ± 0.09
HDL	0.72 ± 0.09	0.85 ± 0.07	0.82 ± 0.25	0.86 ± 0.21*	0.74 ± 0.21**
LDL	0.28 ± 0.08	0.41 ± 0.09	0.37 ± 0.02	0.33.±0.05*	0.24 ± 0.05**

Values are shown as means ± SEM; *P < 0.05 versus STZ group; **P < 0.01 versus STZ group.

**Table 4:** Effect of PIO on GOT, GPT, and LDH.

Groups	GOT (Unit L^−1^)	GPT (Unit L^−1^)	(Unit L^−1^)
Control group	79 ± 3.1	66 ± 3.3	48 ± 7.1
STZ group	288 ± 3.1	159 ± 3.7	316 ± 10.9
PIO (10 mg) group	185 ± 10.2	150 ± 11.3*	310 ± 11.1
PIO (20 mg) group	103 ± 4.0*	129 ± 30.0*	186 ± 10.2*
PIO (30 mg) group	88 ± 4.2*	81 ± 6.1*	77 ± 11.5*

Values are shown as means ± SEM; *P < 0.05 versus STZ group.
